# Broadband terahertz-power extracting by using electron cyclotron maser

**DOI:** 10.1038/s41598-017-07545-6

**Published:** 2017-08-04

**Authors:** Shi Pan, Chao-Hai Du, Xiang-Bo Qi, Pu-Kun Liu

**Affiliations:** School of Electronics Engineering and Computer Science, Peking University, Beijing, 100871 P. R. China

## Abstract

Terahertz applications urgently require high performance and room temperature terahertz sources. The gyrotron based on the principle of electron cyclotron maser is able to generate watt-to-megawatt level terahertz radiation, and becomes an exceptional role in the frontiers of energy, security and biomedicine. However, in normal conditions, a terahertz gyrotron could generate terahertz radiation with high efficiency on a single frequency or with low efficiency in a relatively narrow tuning band. Here a frequency tuning scheme for the terahertz gyrotron utilizing sequentially switching among several whispering-gallery modes is proposed to reach high performance with broadband, coherence and high power simultaneously. Such mode-switching gyrotron has the potential of generating broadband radiation with 100-GHz-level bandwidth. Even wider bandwidth is limited by the frequency-dependent effective electrical length of the cavity. Preliminary investigation applies a pre-bunched circuit to the single-mode wide-band tuning. Then, more broadband sweeping is produced by mode switching in great-range magnetic tuning. The effect of mode competition, as well as critical engineering techniques on frequency tuning is discussed to confirm the feasibility for the case close to reality. This multi-mode-switching scheme could make gyrotron a promising device towards bridging the so-called terahertz gap.

## Introduction

Terahertz (THz) sources mainly include two families, namely optical sources^1^ and electronic sources^2^. Most of the electronic sources are developed from microwave band towards THz band. The gyrotron, as a member of vacuum electronic sources, demonstrates high efficiency and high power capability in THz band^2–9^. A gyrotron employs a relativistic helical electron beam to interact with electromagnetic (EM) wave based on the instability of Electron Cyclotron Maser (ECM). Beam-wave interaction with phase-space azimuthal bunching extracts kinetic energy from electron beam and transfers it to EM radiation^3^. A gyrotron oscillator mostly operates in a single rotating waveguide mode. It produces coherent high-power output on a fixed frequency or in a limited tunable bandwidth. Gyrotrons with high-power capability are important to fusion plasma heating in International Thermonuclear Experimental Reactor (ITER) projects^9–11^. As to other applications, such as high resolution radar^12^, non-destructive inspection^13^ and dynamic nuclear polarization enhanced nuclear magnetic resonance (DNP-NMR)^14–16^, gyrotrons with coherent frequency tuning are more favored.

Frequency-tunable gyrotrons have previously been investigated in detail^14–24^. Generally speaking, there are three kinds of frequency-tunable gyrotrons. The first is the discrete-frequency step-tunable gyrotron^18–21^. University of Fukui produced Gyrotron FU Series which radiated on dozens of discrete frequencies between 38 GHz to 889 GHz^19^. The frequency tuning range of the first kind of gyrotron appears to be wide but discrete. The second is the continuously frequency-tunable open-cavity gyrotron^14–16^. The magnetic tuning via altering magnetic field strength *B* and electronic tuning via altering accelerating voltage *V* are normally applied, and frequency tuning bandwidth is about 1 GHz level. Due to the inherent high-Q property of circuit, further extending of the frequency tuning range becomes challenging. In comparison with the gyrotrons above, the third kind of gyrotron, i.e. gyrotron backward-wave oscillator (gyro-BWO) does demonstrate impressive continuous broadband frequency tuning^3^. Other than extensive investigations on the upstream-output gyro-BWO^3^, T. H. Chang *et al*. carried out a downstream-output gyrotron experiment by employing backward-wave interaction in a conventional open-cavity circuit^22^. The experiment generates a 6-GHz tuning range on the TE_1,2_ mode, which preliminarily demonstrates the competence of continuous broadband tuning. They further investigated an open-cavity scheme tapered at the upstream end to improve the interaction efficiency^23^. This special design provides a continuous tuning bandwidth of 6.9 GHz and efficient output over 25% for 0.2 THz TE_0,2_ mode gyrotron. On this basis, C. H. Du *et al*. proposed a pre-bunched circuit to realize a 10-GHz continuous tuning range^24^. Such pre-bunched interaction compresses the energy-modulation length and extends the energy-releasing length of the beam-wave interaction process, and finally demonstrates simultaneous advantages of broadband tuning and high efficiency.

**Figure 1:**
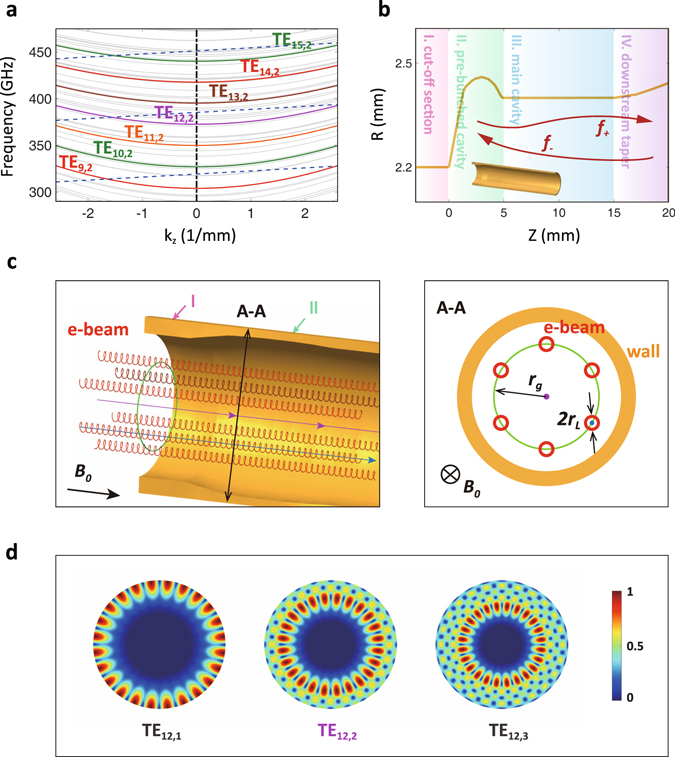
Dispersion and ECM schematic in the pre-bunched structure. (**a**) The cold dispersion relation of multi-mode switching. The colorful curves represent operating modes, and the grey curves represent potential competing modes. The dashed lines represent fundamental harmonic helical electron beam during magnetic tuning. The operating points of concerned backward-wave interaction is located at the left dispersion plane. (**b**) The profile of the pre-bunched backward-wave interaction circuit in gyrotron with four parts. *f*_+_ and *f*_*−*_ indicates forward wave and backward wave, respectively. (**c**) A schematic diagram of the electron movement trajectory in gyrotron. Different electron guiding centers are distributed in the green circles which heads along the blue arrows. Electrons orbit along the guiding centers by spiral movement. (**d**) The normalized horizontal electric field distribution of representative circular waveguide TE_12,1_, TE_12,2_ and TE_12,3_ mode.

In order to achieve continuous broadband THz radiation in a step-tunable gyrotron, this paper proposes a scheme based on magnetic-field-controlled multi-mode switching in backward-wave interaction. A 100-GHz level tuning range is theoretically obtainable. As the cold beam-wave dispersion relation shown in Fig. 1a, the high-order-mode system provides abundant mode spectrum resources that can be explored for multi-mode switching. A pre-bunched circuit shown in Fig. 1b is used to extend the tuning range of each mode. The advantages of step-tunable gyrotrons and gyro-BWOs are directly combined together for multi-mode switching and frequency sweeping. Finally, detailed discussions about mode competition and critical engineering techniques ensure the feasibility of broadband frequency sweeping and provide guidance for future proof of principle experiment.

## Results

### Tuning mechanism of broadband radiation

Here the basic principle is that the ECM system (Fig. 1c) is treated as an extractor of the electron cyclotron frequency. Theoretically speaking, when the beam-wave synchronizing condition is satisfied with frequency *ω* following the equation (1), the kinetic energy of the electron beam with specific cyclotron frequency *Ω*_*e*_ can be transferred into EM wave,

1 $$\omega \approx {k}_{z}{v}_{z}+s{{\rm{\Omega }}}_{e}$$

where *k*_*z*_ is the wave number, *v*_*z*_ is the longitudinal electron velocity, and *s* is the harmonic number. In a cylindrical open cavity, the inherent high-Q standing-wave modes possess discrete longitudinal indexes, which makes *k*_*z*_ replaced by discrete wavenumbers as,

2 $${k}_{z}\approx n\pi /{L}_{eff},\quad n=1,2,3,\mathrm{...}$$

where *L*_*eff*_ is the effective interaction length of the circuit. Accordingly, a gyrotron using a traditional open cavity tends to generate discrete frequencies on specific operating modes in the shape of standing wave. In our proposed scheme, the waveguide gyrotron circuit conveys a travelling wave with continuously varying wavenumber *k*_*z*_. Obviously, according to equation (1), such self-adjusting wavenumber *k*_*z*_ together with magnetic-field-controlled changing *Ω*_*e*_ would help to generate continuous tuning wave frequency *ω*. Consequently, the travelling-wave circuit features broader frequency tuning range than standing-wave circuit. One factor limiting the continuous tuning bandwidth of conventional gyrotron is that the closed sidewall of the interaction circuit quantizes/discretizes the mode spectrum, and each mode only meets equation (1) in a limited band. Supposing a sequential set of modes meet equation (1) during the course of changing *Ω*_*e*_ and each mode generates a reasonable band, an overall broadband tuning range can be approximately bridged.

Compared with traditional frequency-tunable schemes, this scheme includes two significant advancements. Firstly, the feasibility of the backward-wave interaction by using a very high-order whispering-gallery mode is theoretically confirmed. The effective beam-wave interaction and reasonable broadband tuning under single-mode operation are achieved. In consideration of suppressing mode competition and ohmic loss in THz band, whispering-gallery TE_m,2_ modes are selected as the operating modes. Secondly, the single-mode tuning develops into multi-mode switching. By increasing the magnetic field from lower to higher strengths, the gyrotron sequentially switches between a set of nearby whispering-gallery modes to achieve impressive broadband tuning capability. The multi-mode switching is confirmed by both frequency-domain and time-domain investigations.

In this paper, the magnetic tuning is employed for broadband radiation. Considering another conventional frequency tuning method i.e. electronic tuning, magnetic tuning is determined to be used for three reasons^25^. Firstly, the *B* is proportional to *Ω*_*e*_. *V* is inversely proportional to *Ω*_*e*_, and indirectly changes *Ω*_*e*_ by a small tuning to relativity factor *γ*. So, magnetic tuning is more sensitive and effective for broadband tuning^25, 26^. Secondly, frequency tuning by changing voltage always takes steerable mode transformation or engineering control as the principal objective^27, 28^, which goes against our original intention of broadband tuning. Thirdly, electronic tuning could easily cause arcing in the high voltage supply of electron gun and excessive variation of electron beam parameters, which deteriorates the stabilization of gyrotron system. When the tuning efficiency, practical purpose, and operational security are all synthesized, magnetic tuning is found to be more appropriate to the multi-mode frequency tuning scheme.

### Broadband single-mode operation

The designed frequency-tunable gyrotron employs a pre-bunched cavity loaded into the traditional open-cavity interaction circuit. For a given mode, the upstream pre-bunched section introduces additional phase difference between the forward-wave and backward-wave components. Here EM wave performs more like a travelling wave rather than a high-Q standing wave^24^. Such pre-bunched circuit is potential to suppress gyromonotron oscillations (Fig. 1b). The upstream cut-off section can suppress EM wave leaking towards the electron gun. The forward wave brings reflected backward-wave energy to the downstream section. As a premise of multi-mode broadband tuning, each operating mode should improve efficiency and extend tuning range as much as possible. More detailed physics about ECM in pre-bunched circuit was addressed in early studies^23, 24^.

To achieve sequential mode switching, high-order whispering-gallery modes are selected for operation. Whispering-gallery modes possess excellent mode selectivity and high power capacity. Furthermore, in order to decrease ohmic loss and improve mode homogeneity, high azimuthal index modes are selected. When azimuthal indexes are same, TE_m,2_ mode gets a greater distance between the peak electric field and waveguide wall than TE_m,1_ mode. Meanwhile, compared with TE_m,3_ mode, TE_m,2_ mode is easier to suppress competing modes. Therefore, TE_m,2_ mode becomes a reasonable choice for multi-mode switching operation (Fig. 1d). Investigation reveals that operating parameters of the THz frequency-tunable gyrotron have no essential effect on broadband output. In this paper, we follow standard practices and experimental requirements, and propose the optimized parameter lists as shown in Table 1.

**Table 1:** Operating parameters of the THz frequency-tunable gyrotron.

mode	*TE*_*m,2*_, (*m* = 9, 10…, 15)
harmonic number	*s* = 1
accelerating voltage	*V* = 4 kV
current	*I*_*b*_ = 2 A
pitch factor	*α* = *v*_*⊥*_/*v*_*z*_ = 1.5
velocity spread	*Δβ*_*z*_ = *Δv*_*z*_ */v*_*z*_ = 6%
guiding center radius	*r*_*g*_ = 1.68 mm
larmor radius	*r*_*L*_ = 13.283 μm
Cavity radius in main cavity	*r*_*ω*_ = 2.4 mm

**Figure 2:**
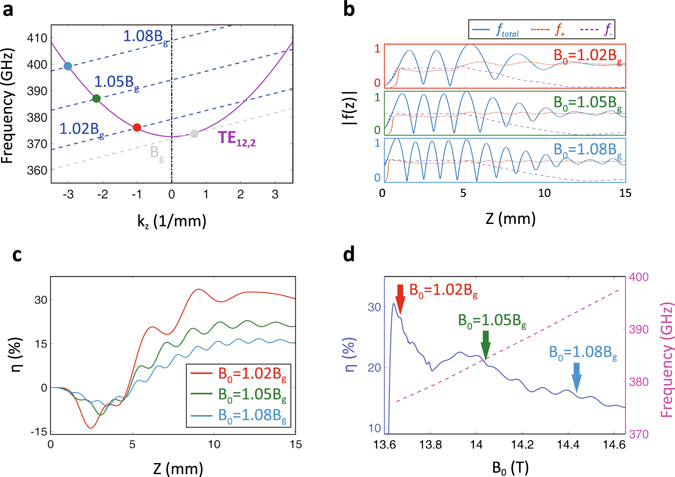
Analysis of broadband single-mode operation. (**a**) The cold dispersion relation between the TE_12,2_ mode and three representative fundamental harmonic helical electron beam modes, corresponding to magnetic field strengths of 1.02*B*_*g*_, 1.05*B*_*g*_ and 1.08*B*_*g*_, respectively. *B*_*g*_ is the magnetic field strength under tangential incidence with determined electron beam parameters. (**b**) The comparison of field profiles of the TE_12,2_ mode in different magnetic field strengths. *f*_*total*_ indicates the total wave, while *f*_+_ and *f*_*−*_ indicate the forward wave and backward wave, respectively. (**c**) The evolutionary process of electron beam efficiencies for three axial modes of the TE_12,2_ mode. (**d**) The output efficiency in quasi-steady state and the continuous tuning range of the TE_12,2_ mode.

The design takes the TE_12,2_ mode as the center mode. With existing parameters, we get the cold dispersion relation of the TE_12,2_ mode and three representative helical electron beam modes as shown in Fig. 2a. The dashed lines represent the electron beam modes under three magnetic field strengths, corresponding to 1.02*B*_*g*_, 1.05*B*_*g*_ and 1.08*B*_*g*_, respectively, where *B*_*g*_ is the magnetic field strength where the electron beam line gets tangent to the TE_12,2_ mode line. The operating point of the EM wave is just around the cross point, which is closer to the cutoff point. As increasing the magnetic field strength, the beam-wave cross point shifts from the right-half forward-wave plane to the left-half backward-wave plane. Obviously, the left-side points correspond to a broader range of operating frequencies. From frequency-domain steady-state calculation^3, 24^, Fig. 2b illustrates the axial mode profiles of the forward wave, the backward wave and the total wave. The results demonstrate that the backward wave is aroused in the middle of circuit and reinforced towards upstream until it grows into the highest strength around the pre-bunched cavity. Next, the backward wave encounters the upstream end with reflection, and rapidly transfers energy into the forward wave. This kind of inherent feed-back mechanism of backward wave is the cause of oscillation. Finally, the backward-wave power is brought out of the cavity by the downstream forward wave.

A group of total-wave fluctuation corresponds to a specific axial mode. As increasing the magnetic field strength, the axial index is boosted accordingly. Figure 2c presents the evolutionary process of electron beam efficiencies for three axial modes. The electron beam absorbs a small amount of energy from EM waves in pre-bunched cavity for backward-wave modulation, and then releases incremental energy in adjacent uniform main cavity. Note that the strongest field of TE_12,2_ mode does not approach to the waveguide wall, the ohmic loss efficiency becomes steady about 3%. The difference between electron beam efficiency and ohmic loss efficiency is the output efficiency. When the magnetic field strength changing from 1.02*B*_*g*_ to 1.08*B*_*g*_, the output efficiency in quasi-steady segment decreases from around 28% to under 15% (Fig. 2d). Thus the beam-wave energy exchange fades out in deep backward-wave interaction, and a higher frequency with a larger *k*_*z*_ demonstrates severe fluctuations on the power profile due to forward-wave modulation^23, 24^.

The frequency-domain single-mode simulation also indicates that the TE_12,2_ mode can generate radiation in a broad bandwidth of 17 GHz between 376 GHz and 393 GHz when the magnetic tuning sweeps over 0.8 T from 1.02*B*_*g*_ to 1.08*B*_*g*_ (Fig. 2d). Under multi-mode condition, this tuning range may be split because of competing modes nibbling frequency space. However, an obvious advantage is that there is a quasi-linear correlation between the frequency response and magnetic field strength, disagreeing with intuitively predicted hyperbolic relation as reported in previous study^14, 15^. This phenomenon can be explained from two aspects. Firstly, oscillation in backward-wave region is well above the cutoff frequency and normally with high wave number (|*k*_*z*_| ≫ 0). Secondly, the pre-bunched cavity provides additional space for interaction adjustment. For example, with low magnetic field strength, the EM energy is mostly confined in the main cavity. With high magnetic field strength, the effective backward-wave interaction region is extended, covering parts of upstream pre-bunched cavity and downstream taper. Such interaction adjustment leads to compensation to the frequency drift due to the Doppler shift *k*_*z*_ *v*_*z*_.

### Multi-band radiation by sequential-mode-switching operation

**Figure 3:**
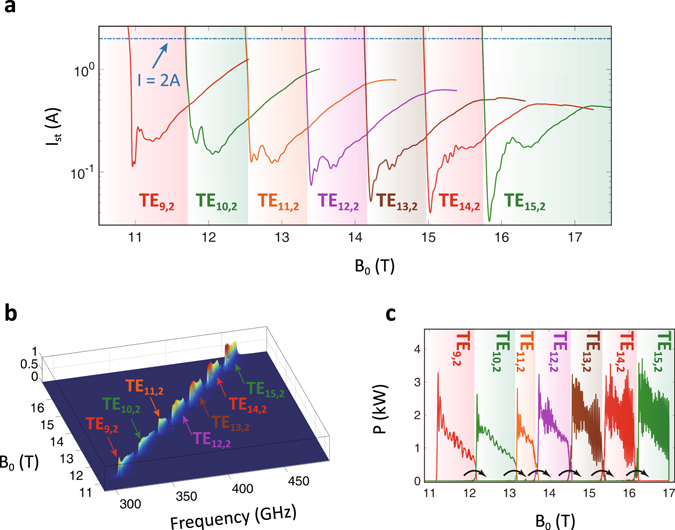
Multi-band radiation by mode switching. (**a**) The start-oscillation current curves of operating modes in different magnetic field strengths. The working current is selected as 2 A. (**b**) The frequency spectrum (Values in z axis represent the normalization of energy distribution) and (**c**) output power from the time-domain multi-mode simulation.

Based on nonlinear self-consistent frequency-domain theory^3, 24^, the calculated start-oscillation currents basically reflect the intrinsic properties of operating modes in the designed circuit. Assuming constant electron beam parameters, the start-oscillation currents *I*_*st*_ of seven operating modes are shown in Fig. 3a. According to previous study about the backward-wave interaction circuit, a mode with lower *I*_*st*_ takes high priority of dominating the oscillation and suppressing other modes^22–24^. Here each current curve goes down first and then rebounds with increasing magnetic field strength. The lowest peak of the *I*_*st*_ curve corresponds to the gyromonotron state in the main cavity. The backward-wave state takes places after the second depression, which is also the mode-transitional zone. When the gyrotron working current is selected as 2 A, the start-oscillation currents of all modes are mostly below the working current. Consequently, each mode can be excited and gets dominating access to beam-wave interaction in a certain range of *B* with lowest *I*_*st*_.

Electrical length *L/λ*, as the ratio between the circuit length *L* and wavelength *λ*, is an important factor to limit the tuning bandwidth. Effective electrical length of the circuit can influence start-oscillation modes. As illustrated in Fig. 3a, only TE_m,2_ modes (*m* = 9, 10, …, 15) are selected as the representative operating modes. For the mode with a smaller azimuthal index *m*, lower frequency results in a shorter electrical length, a smaller Q factor, and lower energy conversion efficiency. Finally, an over-low-order mode never oscillates. Continuous magnetic-controlled frequency tuning range will be split by lower-order mode due to short electrical length. On the other hand, once *m* and operating frequency become higher, the increased electrical length will reduce the *I*_*st*_. Increased electrical length compels the pre-bunched cavity to be relatively enlarged and regarded as a main cavity to excite powerful gyromonotron state. The depression and rebound in the front section of each high-order-mode curve in Fig. 3a get severer. The overlap of *I*_*st*_ curves is gradually serious as magnetic field increasing which induces drastic competition between operating modes. As a result, appropriate electrical lengths of the interaction circuit determine available modes. This is an important reason why we cannot infinitely extend frequency tuning range by multi-mode switching.

The multi-mode switching is tested based on time-domain theory^3, 24, 29^. Regarding TE_9,2_, TE_10,2_, …, TE_15,2_ as operating modes, the frequency spectrum without considering competing modes is presented in Fig. 3b. The summary of tuning parameters and output parameters is shown in Table 2. Weak lossy materials are loaded in pre-bunched cavity to prevent its cavity-oscillation effect and improve the modulation competence for EM wave energy^23, 24^. The magnetic tuning process from 10.71 T to 17.00 T corresponds to a linear variation in time domain. The gyrotron consecutively switches from a low-order mode to a high-order mode, and output frequency spectrum almost covers the band between 309 GHz and 462 GHz by seven modes during the effective magnetic tuning range of 11.18 T to 17.00 T. The intrinsic dispersion provides a limited region for gyromonotron state. In addition, during the process of increasing magnetic field, the established backward-wave oscillating mode is easier to extract electron beam energy than a latter gyromonotron-state mode. So, each mode mostly operates in backward-wave state. Obviously, start-oscillation states of continuous frequency tuning in Fig. 3b are consistent with the prediction from start-oscillation currents in Fig. 3a. The result also confirms the consistency and validity of the frequency-domain single-mode and the time-domain multi-mode analyses.

**Table 2:** Tuning parameters and output parameters in ideal multi-band radiation.

linear magnetic tuning range	10.71–17.00 T
effective magnetic tuning range	11.18–17.00 T
quasi-continuous frequency tuning range	309–462 GHz
output power	0.6–2 kW
output efficiency	7.5% − 25%

The frequency tuning spectrum for seven operating modes is quasi-continuous, however the output power (Fig. 3c) is continuous. It mainly ranges between 0.6 kW and 2 kW, corresponding to 7.5% to 25% efficiency, which is a little lower than that from frequency-domain single-mode simulation. There are two reasons leading to this phenomenon. One is that perfectly matched layer (PML) absorbing boundary condition used in time-domain simulation induces weak reflections. For frequency-domain simulation, ideal outgoing-wave boundary condition induces no reflection. The other is that the magnetic field strength in time-domain simulation varies relatively fast with time due to limited calculation resource, which counts against establishing absolutely stable operation and sufficient energy conversion. In practical process, each mode will have more enough time to start oscillation. In addition, the output power demonstrates fluctuations for three reasons. The specific reasons are slightly different for low-order modes and high-order modes. Firstly, limited number of macro-particles is the common reason about the setting of simulation conditions. Increasing the macro-particle number by several times, the output power, especially in low frequency, filters out a lot of fluctuations. However, the time consuming of simulation linearly increases accordingly. Secondly, two intrinsic factors affect low-order modes and high-order modes, respectively. For low-order modes, periodic absorbing and releasing of forward wave energy is another important reason for power fluctuation (Fig. 2b), which cannot be avoided in downstream-output structure^24^. For high-order modes, backward-wave axial-mode competition is the primary cause to fluctuation, which is a natural phenomenon in a multi-mode system. This problem is related to parameter setting, mode selection and interaction structure design. Relevant theoretical exposition is shown in supplementary material.

## Discussion and Conclusions

There are three aspects imposing potential limitations to available ECM tuning range. Firstly, the same interaction circuit demonstrates different effective electrical lengths for sequential whispering-gallery modes. Such difference results in limited number of available modes in a given circuit. Secondly, competing modes may induce additional disturbance to break off continuous tuning range. Thirdly, present engineering techniques such as controlling variation of electron beam parameters may not meet the broadband tuning requirements absolutely. In the following we will discuss the latter two points for the feasibility consideration of broadband THz frequency extracting of ECM.

### Analysis of mode competition

**Figure 4:**
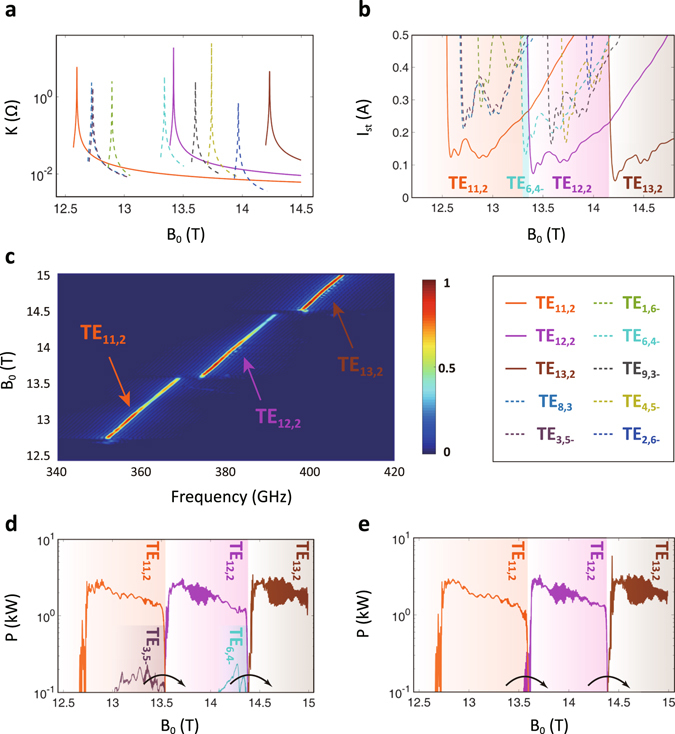
Multi-band radiation taking account of mode competition. (**a**) The beam-wave coupling impedances *K* of TE_11,2_, TE_12,2_ and TE_13,2_ modes in solid lines, as well as internal threatening competing modes in dashed lines. The counter-rotating mode is with a minus while the co-rotating mode has no specific sign. (**b**) The start-oscillation current curves of TE_11,2_, TE_12,2_ and TE_13,2_ modes, as well as internal competing modes. (**c**) The frequency spectrum and (**d**) output power generated by TE_11,2_, TE_12,2_ and TE_13,2_ modes and internal competing modes. (**e**) The output power of TE_11,2_, TE_12,2_ and TE_13,2_ modes without mode competition. It takes the same magnetic field sweeping rate as that in competition condition.

To simulate realistic mode excitation, a scaled situation, involving mode switching among the TE_11,2_, TE_12,2_ and TE_13,2_ mode, is displayed. Influence of all the threatening competing modes in this band will be investigated in this part. In the first place, the concept of the coupling impedance *K* is applied to briefly evaluating the beam-wave coupling strength in a referential uniform cylindrical system. The larger *K* is, the easier beam-wave interaction happens. Electron beam and EM wave characteristics, especially frequency and phase velocity, are simultaneously included in *K*. The analytical expressions are given as^30^

3 $$K=\mu {H}_{sm}\frac{{r}_{L}^{2}}{{s}^{2}}\frac{\omega }{{k}_{z}{K}_{\perp }}$$

4 $${K}_{\perp }=\pi {r}_{\omega }^{2}[1-\frac{{m}^{2}}{{({k}_{\perp }{r}_{\omega })}^{2}}]{J}_{{\rm{m}}}^{2}({k}_{\perp }{r}_{\omega })$$

where *H*_*sm*_ is the coupling coefficient, *k*_⊥_ and *k*_*z*_ are the transverse and longitudinal wave numbers, *J*_*m*_ is the Bessel function of order *m*. Substituting the beam-wave equations in ref. 1 into these two expressions, the relationship between the magnetic field strength and the coupling impedance is obtained as shown in Fig. 4a. Each peak corresponds to the singular cutoff point of a mode, where it assumes *k*_*z*_ = 0. Comparing the absolute values of *K* for different modes, we can demonstrate the quantitative coupling strengths under different magnetic field strengths and operating frequencies. Anomalous intersections in impedance curves show that competing modes in the near-cutoff region are very dangerous for whispering-gallery modes.

The start-oscillation current curves bring further comprehensive consideration of nonlinear interaction (Fig. 4b). The dashed lines represent the competing modes while the solid lines represent the operating modes. Each of the three operating modes can maintain the lowest start-oscillation current region, apart from a small region of the TE_6,4-_ mode as magnetic field around 13.3 T. Considering the general principle that modes with lowest start-oscillation current are always predominant modes in a travelling-wave circuit^22–24^, mode competition is discovered to be possible but relatively feeble.

The time-domain analysis with mode competition gives a straightforward result as shown in Table 3 and Fig. 4c. In case of exciting the TE_11,2_ mode first, the TE_12,2_ and TE_13,2_ modes govern over the circuit successively. The spectrum of TE_11,2_, TE_12,2_ and TE_13,2_ modes are 351–369 GHz, 373–392 GHz and 398–410 GHz, respectively. Compared with the ideal operation in Fig. 3b, TE_11,2_ and TE_13,2_ modes separately extend frequency responding range forwards and backwards because of no front and back extrusion from other modes. The mode selection, automatic switching and wide-multi-band radiation are all feasible under mode competition condition. The initial oscillation frequency for TE_12,2_ mode is 373 GHz, agreeing well with theoretical prediction in cold dispersion relation (Fig. 2a). Magnetic field strengths in two dynamic mode switching processes are located in the predicted ranges based on start-oscillation current curves. Therefore, the consistency between cold-field stable-state calculation, frequency-domain calculation and time-domain analysis confirms the reliability of investigation again.

**Table 3:** Tuning parameters and output parameters in mode competition.

linear magnetic tuning range	12.45–14.99 T	
continuous frequency tuning range	TE_11,2_ mode	351–369 GHz
	TE_12,2_ mode	373–392 GHz
	TE_13,2_ mode	398–410 GHz
output efficiency	15% − 25%	

Figure 4d presents continuous output power with mode competition, which is rather higher than that in ideal condition (Fig. 3c). This is because that here the lower magnetic field sweeping rate provides more enough time for operating modes to realize sufficient energy conversion. To eliminate the difference of simulation setting, we conduct a comparative analysis (Fig. 4e). The results indicate that mode competition does not decrease the output power of operating modes, but only slightly alters the magnetic field strength of mode-switching point.

### Critical engineering techniques

To cover the basics for future proof of principle experiment, we briefly discuss two limitations to the development of gyrotron system. One point is that, parameters of general electron gun are difficult to maintain constant values during magnetic tuning^31, 32^. In supplementary material, we take magnetic injection gun (MIG) as an example to illustrate that relatively stable electron beam parameters are available by using auxiliary coils. A latest paper reveals that slight variation of electron beam parameters does not influence the essence of frequency tuning^29^, which enhances the feasibility of multi-mode frequency tuning. The other point is that, if pulse magnet is employed, the start and stop of high pulse magnet will produce a powerful induction current. Normal copper circuit is easy to be intensively extruded and deformed by a force from the induction current^5, 6^. To address this issue, electroplating or spraying techniques can be used to restructure a more robust composite circuit.

To conclude, this paper proposes a scheme of broadband THz radiation source based on multi-mode switching gyrotron. Tuning mechanism and simulation results confirm that the source is potential to generate broadband tuning range on the order of 100-GHz level, boosting about two order of magnitudes higher than the state-of-art techniques. Discussion about mode competition and engineering techniques provides a powerful evidence for the scheme feasibility. The proposed ECM THz extractor would become a general solution to generate high-power broadband coherent radiation from THz helical electron beam. That is to say, as long as helical electron beam exists, coherent THz-wave would be excited.

## Methods

### Frequency-domain theory and time-domain theory

In frequency-domain theory and time-domain theory, the beam-wave interaction equations derived from Maxwell’s equations, and the electron dynamics equations describing the movement of electron beam are employed together to simulate self-consistent beam-wave interaction. Frequency-domain calculation with outgoing wave boundary condition concentrates on one EM waveguide mode interacting with electron beam in stable state^3, 24, 29^. Time-domain calculation with PML absorbing boundary condition demonstrates the time-varying beam-wave energy exchange and could present time-varying multi-mode construction and the mode competition evolvement^3, 24, 29^.

### Macro-particle assumption and division of time segments

As a matter of fact, the electron trajectory in gyro-devices is similar to the moon movement in solar system. An electron (like ‘moon’) orbits the guiding center (like ‘Earth’) in Larmor circle along the magnetic flux line, while the guiding center orbits the cavity transverse center (like ‘Sun’). For simplifying the calculation model, a macro-particle with same electric quantity would substitute for several electrons in Larmor circle. Next, divided emission time segments of macro-particles is applied to approximate successive electron beam/stream. Under the premise of convergence precision, the sparse time-domain and space-domain meshes in this way are beneficial to improve simulation efficiency.

## Electronic supplementary material

Supplementary information
