# Supplementary material for: Broadband terahertz-power extracting by using electron cyclotron maser
Source: Sci Rep. 2017 Aug 4;7:7265. doi: 10.1038/s41598-017-07545-6 (PMC5544717; doi:10.1038/s41598-017-07545-6)
Supplement: Supplementary file 1 — Supplementary information [file 41598_2017_7545_MOESM1_ESM.pdf]

Supplementary material for "Broadband terahertz-power extracting by using electron cyclotron maser"

Shi Pan, Chao-Hai Du*, Xiang-Bo Qi, Pu-Kun Liu*

School of Electronics Engineering and Computer Science, Peking University, Beijing 100871, P. R. China

duchaohai@pku.edu.cn, pkliu@pku.edu.cn

Appendix I: Frequency-domain theory

Here we present frequency-domain interaction between the electron beam and the TE modes existing in a circular waveguide (Fig. s1). Firstly, the derivation from frequency-domain Maxwell equations is a departure for electron acting on EM wave. We can get the following expression about the horizontal electric field component by using cold field assumption (assuming horizontal field components in gyrotron similar to those in the passive situation) and variable separation method.

$$\left(\frac{\partial^2}{\partial z^2}+k_z^2\right)\mathrm{Im}\left(\vec{E}_\perp\right)=\omega\mu_0\vec{J}_\perp \tag{s1}$$

Substituting horizontal electric field components into equation (s1) and operating $\left(\frac{\omega}{2\pi}\right)\int_0^{2\pi/\omega}dt\int_0^{r_w}\int_0^{2\pi}\cdot\frac{\vec{E}_\perp^*}{f^*(z)}rdrd\phi$ on both sides of equation (s1), it yields following beam-wave interaction equation.

$$\left(k_z^2+\frac{d^2}{dz^2}\right)f(z)=j\frac{2}{\omega\mu_0 f^*(z)}\frac{|k_\perp|^2}{G_{mn}}\left(\frac{\omega}{2\pi}\right)\int_0^{2\pi/\omega}\int_0^{r_\omega}\int_0^{2\pi}\left(\vec{J}_\perp\cdot\vec{E}_\perp^*\right)rdrdtd\phi \tag{s2}$$

Where $f(z)=f_+(z)e^{-jk_zz}+f_-(z)e^{jk_zz}$ denotes the field amplitude along the z axis, $G_{mn}=\pi r_\omega{}^2\left(1-\frac{m^2}{k_\perp^2 r_\omega{}^2}\right)J_m^2\left(k_\perp r_\omega\right)$ and $k_\perp^2=k^2-k_z^2$.

Returning to Maxwell equations again, the general field expressions of circular-waveguide TE_{mn} modes are given by the real part of the following expressions.

$$\begin{cases} H_z=f(z)J_m\left(k_\perp r\right)\mathrm{e}^{j(\omega t-m\phi)} \\ E_\perp=\frac{1}{k_\perp^2}j\omega\mu\vec{e}_z\times\nabla_\perp H_z \\ H_\perp=\frac{1}{k_\perp^2}\frac{\partial}{\partial z}\nabla_\perp H_z \end{cases} \tag{s3}$$

For convenience to the nonlinear theory[1] in beam-wave interaction system, the field expressions above

can also be expanded into Guiding Center Coordinate (GCC) system (from <r, ϕ, z> to <r_L, θ, z>). Specific changes are as follows.

$$\begin{cases} H_z = f(z)\sum_{s=-\infty}^{\infty} J_s(k_\perp r_L) J_{m-s}(k_\perp r_g) e^{j\omega t - js\theta - j(m-s)\phi_g} \\ E_{r_L} = -\frac{\omega\mu_0}{k_\perp^2 r_L} f(z)\sum_{s=-\infty}^{\infty} sJ_s(k_\perp r_L) J_{m-s}(k_\perp r_g) e^{j\omega t - js\theta - j(m-s)\phi_g} \\ E_\theta = \frac{j\omega\mu_0}{k_\perp} f(z)\sum_{s=-\infty}^{\infty} J_s'(k_\perp r_L) J_{m-s}(k_\perp r_g) e^{j\omega t - js\theta - j(m-s)\phi_g} \\ H_{r_L} = \frac{1}{k_\perp} f'(z)\sum_{s=-\infty}^{\infty} J_s'(k_\perp r_L) J_{m-s}(k_\perp r_g) e^{j\omega t - js\theta - j(m-s)\phi_g} \\ H_\theta = \frac{-j}{k_\perp^2 r_L} f'(z)\sum_{s=-\infty}^{\infty} sJ_s(k_\perp r_L) J_{m-s}(k_\perp r_g) e^{j\omega t - js\theta - j(m-s)\phi_g} \end{cases} \tag{s4}$$

Then the beam current can be represented by macro-particles as,

$$\vec{J} = C\sum_{i=1}^{N} W_i \frac{1}{r_i}\delta(r - r_i)\delta(\varphi - \varphi_i)\delta(z - z_i)\vec{v}_i \tag{s5}$$

We eventually obtain the impact of electrons on EM wave as,

$$\left(k_z^2 + \frac{d^2}{dz^2}\right) f(z) = -2\frac{|I_b| k_\perp}{G_{mn}} \sum_{i=1}^{N} W_i \frac{\vec{v}_i}{v_{zi}} \cdot \left[\sum_{s=-\infty}^{\infty} J_s'\left(k_\perp r_{L_i}\right) J_{m-s}\left(k_\perp r_{g_i}\right) e^{j\Lambda_i}\right]^* \tag{s6}$$

where the phase factor is $\Lambda_i = \omega t_i - s\theta_i - (m-s)\phi_{g_i}$.

Secondly, the following equation describes the electron movement state with EM wave and external magnetic field,

$$\frac{d\vec{p}}{dt} = -e\vec{E} - e\vec{v}\times\left(\vec{B} + \vec{B}_0\right) = v_z \frac{d}{dz}\vec{p} \tag{s7}$$

Moreover, the evolution equations of the electron movement state and the guiding center are as follows.

$$\frac{d}{dz}\vec{p} = -P_\theta \frac{d\theta}{dz}\vec{e}_{r_L} + \frac{d}{dz}P_\theta \vec{e}_\theta + \frac{d}{dz}P_z \vec{e}_z \tag{s8}$$

$$\begin{cases} \dfrac{d}{dz}P_z = e\dfrac{v_\theta}{v_z}\mathrm{Re}\left[\dfrac{\mu}{k_\perp}f'(z)\sum_{s=-\infty}^{\infty}J_s'(k_\perp r_L)J_{m-s}\left(k_\perp r_g\right)e^{j\Lambda}\right] \\ \qquad -geB_0\dfrac{v_\theta}{v_z}\left[r_L + r_g\cos\left(\theta-\phi_g\right)\right] \\ \dfrac{d}{dz}P_\theta = -\dfrac{e}{v_z}\mathrm{Re}\left[\dfrac{\mu}{k_\perp}\left[j\omega f(z)+v_z f'(z)\right]\sum_{s=-\infty}^{\infty}J_s'(k_\perp r_L)J_{m-s}\left(k_\perp r_g\right)e^{j\Lambda}\right] \\ \qquad +geB_0\left[r_L + r_g\cos\left(\theta-\phi_g\right)\right] \\ P_\theta\dfrac{d\theta}{dz} = -\dfrac{e}{v_z}\mathrm{Re}\left[\dfrac{\mu}{k_\perp^2}\sum_{s=-\infty}^{\infty}\left(\dfrac{s\omega}{r_L}f(z)-k_\perp^2 v_\theta f(z)-\dfrac{jsv_z}{r_L}f'(z)\right)J_s(k_\perp r_L)J_{m-s}\left(k_\perp r_g\right)e^{j\Lambda}\right] \\ \qquad +eB_0\dfrac{v_\theta}{v_z}-eB_0 g r_g\sin\left(\theta-\phi_g\right) \end{cases} \tag{s9}$$

$$\begin{cases} \dfrac{dr_g}{dz} = \dfrac{\mu}{B_0 v_z}\mathrm{Re}\left[\begin{array}{l}\dfrac{1}{k_\perp}\displaystyle\sum_{s=-\infty}^{\infty}J_s(k_\perp r_L)J_{m-s}'\left(k_\perp r_g\right)\left(j\omega f(z)+v_z f'(z)\right)e^{j\Lambda} \\ +j\dfrac{1}{2}\displaystyle\sum_{s=-\infty}^{\infty}\left[\begin{array}{l}J_{s-1}(k_\perp r_L)J_{m-s+1}\left(k_\perp r_g\right) \\ -J_{s+1}(k_\perp r_L)J_{m-s-1}\left(k_\perp r_g\right)\end{array}\right]\cdot v_\theta f(z)e^{j\Lambda}\end{array}\right] \\ \qquad -gr_g + gr_L\cos\left(\theta-\phi_g\right) \\ r_g\dfrac{d\phi_g}{dz} = -\dfrac{\mu}{v_z B_0}\mathrm{Re}\left[\begin{array}{l}j\dfrac{1}{k_\perp}\displaystyle\sum_{s=-\infty}^{\infty}\dfrac{m-s}{k_\perp r_g}J_s(k_\perp r_L)J_{m-s}\left(k_\perp r_g\right)\left(j\omega f(z)+v_z f'(z)\right)e^{j\Lambda} \\ +\dfrac{1}{2}\displaystyle\sum_{s=-\infty}^{\infty}\left[\begin{array}{l}J_{s-1}(k_\perp r_L)J_{m-s+1}\left(k_\perp r_g\right) \\ +J_{s+1}(k_\perp r_L)J_{m-s-1}\left(k_\perp r_g\right)\end{array}\right]\cdot v_\theta f(z)e^{j\Lambda}\end{array}\right] \\ \qquad +gr_L\sin\left(\theta-\phi_g\right) \end{cases} \tag{s10}$$

Where P_z and P_θ indicate the components of momentum, and $g = \dfrac{1}{2B_0}\dfrac{dB_0}{dz}$.

Appendix II: Time-domain theory

Equally based on cold field assumption, variable separation method and macro-particle assumption, Time-domain theory is derived from time-domain Maxwell equations. Firstly, we can obtain the following beam-wave interaction equation.

$$\left(\frac{\partial^2}{\partial z^2}-k_\perp^2-\mu_0\varepsilon_0\frac{\partial^2}{\partial t^2}\right)f(z,t) = -\frac{2\left(k_\perp^*\right)^2}{\mu_0^* K_{mn}\dfrac{\partial}{\partial t}f^*(z,t)}\frac{I_b\cdot\Delta t}{N_t\cdot\Delta z}\sum_{i=1}^{N}W_i\delta\left(z-z_i\right)\vec{v}_{\perp i}\cdot\vec{E}_\perp^* \tag{s11}$$

where $K_{mn} = \pi r_w^2\left(1-\dfrac{m^2}{x_{mn}^2}\right)J_m^2\left(x_{mn}\right)$.

The field expressions can be delivered in GCC system.

$$\begin{cases} B_z = \mu_0 f(z,t) \sum_{s=-\infty}^{\infty} J_s(k_\perp r_L) J_{m-s}(k_\perp r_g) e^{-js\theta - j(m-s)\phi_g} \\ E_{r_L} = \frac{j\mu_0}{k_\perp^2 r_L} \frac{\partial}{\partial t} f(z,t) \sum_{s=-\infty}^{\infty} sJ_s(k_\perp r_L) J_{m-s}(k_\perp r_g) e^{-js\theta - j(m-s)\phi_g} \\ E_\theta = \frac{\mu_0}{k_\perp} \frac{\partial}{\partial t} f(z,t) \sum_{s=-\infty}^{\infty} J_s'(k_\perp r_L) J_{m-s}(k_\perp r_g) e^{-js\theta - j(m-s)\phi_g} \\ B_{r_L} = \frac{\mu_0}{k_\perp} \frac{\partial}{\partial z} f(z,t) \sum_{s=-\infty}^{\infty} J_s'(k_\perp r_L) J_{m-s}(k_\perp r_g) e^{-js\theta - j(m-s)\phi_g} \\ B_\theta = \frac{-j\mu_0}{k_\perp^2 r_L} \frac{\partial}{\partial z} f(z,t) \sum_{s=-\infty}^{\infty} sJ_s(k_\perp r_L) J_{m-s}(k_\perp r_g) e^{-js\theta - j(m-s)\phi_g} \end{cases} \tag{s12}$$

Secondly, force analysis of electron can be expressed as,

$$\frac{d}{dt}\vec{p} = \left(-e\vec{E} - e\vec{v} \times \vec{B}\right) - e\vec{v} \times \vec{B}_0 \tag{s13}$$

The evolution equations of the electron movement state and the guiding center are as follows,

$$\frac{d}{dt}\vec{p} = -P_\theta \frac{d\theta}{dt}\vec{e}_{r_L} + \frac{d}{dt}P_\theta \vec{e}_\theta + \frac{d}{dt}P_z \vec{e}_z \tag{s14}$$

$$\begin{cases} \frac{dP_z}{dt} = \mathrm{Re}\left[ev_\theta B_{r_L} - ev_\theta \frac{1}{2}\frac{dB_0}{dz} r_L - ev_\theta \frac{1}{2}\frac{dB_0}{dz} r_g \cos(\theta - \phi_g)\right] \\ \frac{dP_\theta}{dt} = \mathrm{Re}\left[-eE_\theta - ev_z B_{r_L} + ev_z \frac{1}{2}\frac{dB_0}{dz} r_L + ev_z \frac{1}{2}\frac{dB_0}{dz} r_g \cos(\theta - \phi_g)\right] \\ P_\theta \frac{d\theta}{dt} = \mathrm{Re}\left[eE_{r_L} + e(v_\theta B_z - v_z B_\theta) + ev_\theta B_0 - ev_z \frac{1}{2}\frac{dB_0}{dz} r_g \sin(\theta - \phi_g)\right] \end{cases} \tag{s15}$$

$$\begin{cases} \frac{dr_g}{dt} = \mathrm{Re}\left[\begin{array}{l} \frac{\left[E_{r_L} + (v_\theta B_z - v_z B_\theta)\right]\sin(\theta - \phi_g)}{B_0} + \frac{\left[E_\theta + v_z B_{r_L}\right]\cos(\theta - \phi_g)}{B_0} - v_z \frac{1}{2B_0}\frac{dB_0}{dz} r_g \\ -v_z \frac{1}{2B_0}\frac{dB_0}{dz} r_L \cos(\theta - \phi_g) + \frac{P_\theta}{eB_0^2}\frac{dB_0}{dz}\cos(\theta - \phi_g) \end{array}\right] \\ r_g \frac{d\phi_g}{dt} = \mathrm{Re}\left[\begin{array}{l} \frac{\left[E_\theta + v_z B_{r_L}\right]\sin(\theta - \phi_g)}{B_0} - \frac{\left[E_{r_L} + (v_\theta B_z - v_z B_\theta)\right]\cos(\theta - \phi_g)}{B_0} \\ -v_z \frac{1}{2B_0}\frac{dB_0}{dz} r_L \sin(\theta - \phi_g) + \frac{P_\theta}{eB_0^2}\frac{dB_0}{dz}\sin(\theta - \phi_g) \end{array}\right] \end{cases} \tag{s16}$$

Compared with the frequency-domain beam-wave interaction equations, time-domain equations employ the variable t to simulate mode state including start oscillation, stabilization, competition and cooperation.

Appendix III: The merits of whispering-gallery modes operating in THz gyrotron

In this paper, the $TE_{m,2}$ whispering-gallery modes with m>>n are determined as the operating modes. There are three main reasons for this. Firstly, these high-order modes are beneficial to apply a large-aperture cavity and compress ohmic loss in THz range[2]. Secondly, the radial indexes equal to two which guarantees the peak field of EM wave relatively concentrated for effective beam-wave interaction. Also, a great distance between the peak field and waveguide wall can be obtained to avoid over-high ohmic loss (Fig. s2a). Thirdly, the operating modes possess high azimuthal indexes. The other potential competing modes with close eigenvalues usually correspond to lower azimuthal indexes and higher radial indexes. Thus, there is an obvious discrepancy of the strongest field regions between operating modes and competing modes (Fig. s2a-d). Considering the optimized guiding center radius r_g, only the operating modes i.e. $TE_{m,2}$ modes can maintain powerful beam-wave coupling and sufficient beam-wave interaction. This is in favor of compressing mode competition.

Appendix IV: Confirmation for competing modes of $TE_{11,2}$, $TE_{12,2}$ and $TE_{13,2}$ modes

In view of given design parameters in a referential uniform cylindrical waveguide system, we can preliminarily pick out competing modes near the operating modes based on following beam-wave coupling coefficient equation[1],

$$H_{sm} = J_{s-m}^{2}\left(k_{\perp} r_g\right) J_s'^{2}\left(k_{\perp} r_L\right) \quad \text{(s17)}$$

where $k_{\perp} = x_{mn} / r_{\omega}$. In fact, each eigenvalue corresponds to two rotating modes including a co-rotating mode and a counter-rotating mode in cylindrical waveguide. There is a big difference of beam-wave coupling coefficients between these two kinds of modes (Fig. s3). The operating modes possessing high coupling coefficients illustrates that the beam-wave energy exchange is effective. The competing modes have various coupling coefficients. Some modes (in blue) as threatening competing modes are considered to be analyzed, while others (in grey) as weak competing modes are abandoned.

Appendix V: The analysis of output power fluctuation of high-order operating modes

As shown in this paper, there are some fluctuations of output power in multi-mode simulation. For low-order modes, absorbing and releasing of EM wave energy reflected in forward wave is an important reason. But for high-order modes, backward-wave axial-mode competition functions more. Here, we adopt the $TE_{10,2}$ mode and the $TE_{14,2}$ mode on the behalf of low-order and high-order modes to comparatively study this phenomenon. (Generally, the start-oscillation currents we talked about correspond to first-order backward-wave axial mode.) Figure s4 indicates start-oscillation currents of these two operating modes in first-to-third-order backward-wave axial-mode conditions with the same circuit structure and parameters. The magnetic tuning range corresponds to that in multi-mode system in Fig. 3c. For $TE_{10,2}$ mode (Fig. s4a), only the start-oscillation currents of first-order backward-wave axial mode are completely below the working currents. The other high-order backward-wave axial modes have interrupted current curves. So here just feeble backward-wave axial-mode competition happens. As to $TE_{14,2}$ mode (Fig. s4b), almost all start-oscillation currents of the first three backward-wave axial modes are below the working currents. These backward-wave axial modes can be excited, and mutual competition and interplay leads to severely instable output. Next the output powers of the $TE_{14,2}$ mode with different working currents are compared as shown in Fig. s4c-e. As the working current decreasing, fluctuation of output power becomes milder with sacrifice of interaction efficiency and effective magnetic tuning range. Above analysis illustrates that controlling threshold of working current helps to keep down the backward-wave axial-mode competition and maintain stable output power of high-order

operating modes.

Appendix VI: The analysis of variation of electron beam parameters

The classical engineering design of magnet configuration for electron gun is to employ two coils[3-5]. One is the primary coil located around the interaction circuit, while the other is the auxiliary coil located around the electron emitter. When only the primary coil is obtained, the magnetic compression ratio $\varepsilon = B_0/B_{zc}$ does not change no matter how the coil current varies. (B_{zc} and B_0 are the magnetic field strength in emitter and in interaction part, respectively.) Considering the following first order approximate equation[5, 6],

$$B_{zc}{r_c}^2 = B_0\left(r_g^2 - r_L^2\right) \tag{s18}$$

where r_c is the emitter radius, once ε is constant, r_g almost does not change with negligible r_L. Accordingly, α would vary a lot as shown in equation (s19), where η is the charge-to-mass ratio.

$$\alpha = \left(\frac{2\gamma V}{\eta B_0\left(B_0 r_g^2 - B_{zc}{r_c}^2\right)} - 1\right)^{-\frac{1}{2}} \tag{s19}$$

Hence, only one between r_g and α keeps stable in this condition. The auxiliary coil is next applied to avoiding significant fluctuation of electron beam parameters and helping to get a balance of reasonable and acceptable variation for main electron gun parameters in the tuning process. A quarter section of the electron gun schematic diagram to analyze tuning properties is proposed in Fig. s5a. Z axis is the spin axis. The approximate magnetic field distributions of coil 1 and coil 2 are indicated in brown and red curves, respectively. Coil 2 as an auxiliary generates a smaller magnetic field strength than coil 1. The adjustment of the auxiliary magnetic field strength contributes to the slight tuning of B_{zc} and then ε. Calculations are based on the SLAC EGUN code[7]. When electron beam voltage 4kV and current 2A are determined, optimized variation (Fig. s5b) of ε, approximate to a straight line, is obtained as primary magnetic field tuning from 12.5 T to 14.5 T. This is beneficial to stablize parameters. Accordingly, α in Fig. s5c changes between the maximum value 1.50 and the minimum value 1.44. r_g in Fig. s5d is around 1.68 mm with an error rate lower than 4%. The velocity spread $\Delta\beta_z = \Delta v_z/v_z$ in Fig. s5e varies around 6% within an acceptable scope. By using an auxiliary coil, the value of each parameter is almost a constant. In a word, the variation of electron gun parameters in magnetic tuning perhaps deteriorates the gyrotron performance but not destroys the mechanism of multi-mode broadband tuning.

Reference:

1. K. R. Chu. The electron cyclotron maser. *Rev. Modern Phys.* **76,** 489-540 (2004).
2. Y. J. Huang, L. H. Yeh & K. R. Chu. An analytical study on the diffraction quality factor of open cavities. *Phys. Plasmas* **21,** 103112 (2014)
3. A. C. Torrezan *et al*. Continuous-wave operation of a frequency-tunable 460-GHz second-harmonic gyrotron for enhanced nuclear magnetic resonance. *IEEE Trans. Plasma Sci.* **38,** 1150-1159 (2010).
4. C. R. Donaldson *et al.* A cusp electron gun for millimeter wave gyrodevices. *Appl. Phys. Lett.* 96, 141501 (2010).
5. C. P. Yuan, T. H. Chang, N. C. Chen & Y. S. Yeh. Magnetron injection gun for a broadband gyrotron backward-wave oscillator. *Phys. Plasmas* **16,** 073109 (2009).

6. J. M. Baird & W. Lawson. Magnetron injection gun (MIG) design for gyrotron applications. *Int. J. Electronics* **61,** 953-967 (1986).
7. W. Herrmannsfeldt. EGUN: An electron optics and gun design program. Stanford Linear Accelerator Center, Stanford, CA, Tech. Rep. SLAC-0331 UC-28 (1988).

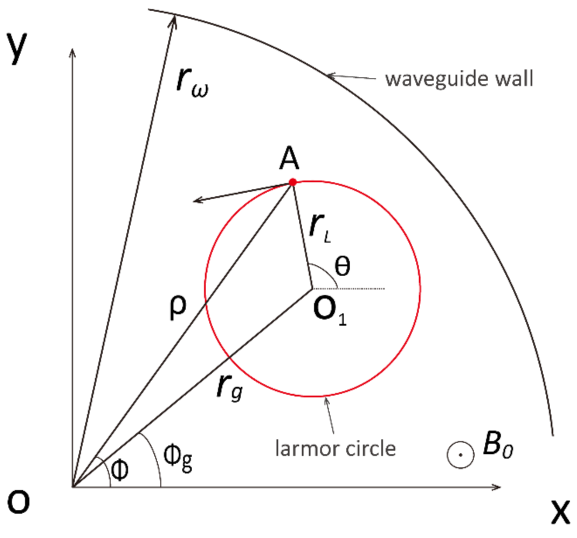

Figure s1. A quarter of cross section in gyrotron interaction circuit.

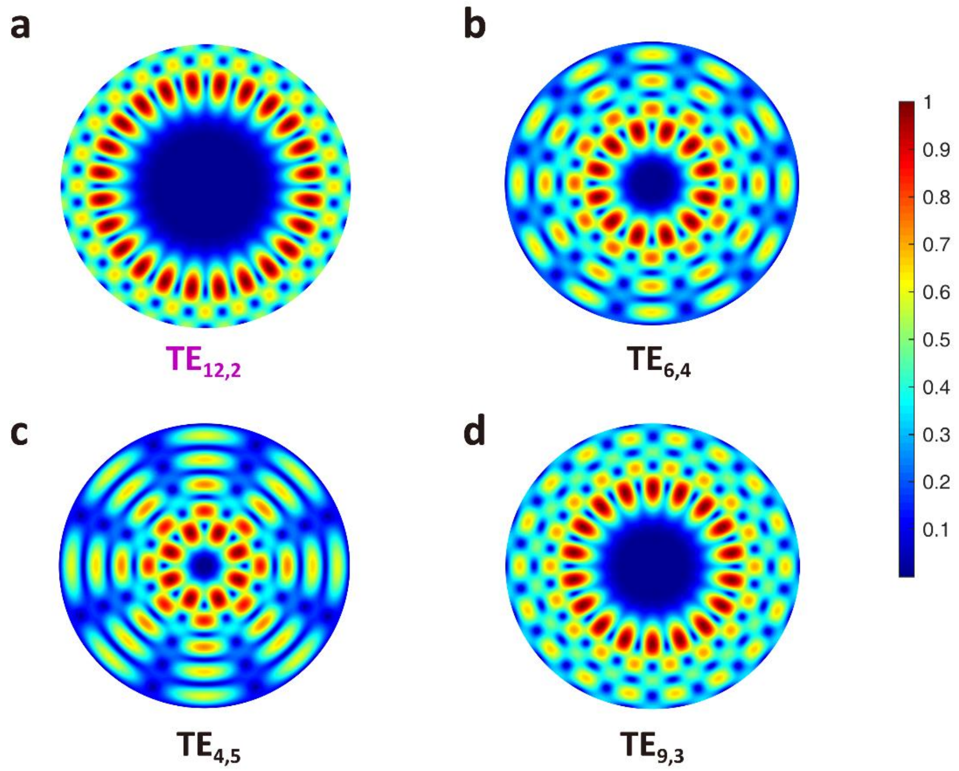

Figure s2. Electric field distribution. The normalized horizontal electric field distribution of cylindrical waveguide **(a)** $TE_{12,2}$ mode and its three nearby competing modes including **(b)** $TE_{6,4}$ mode, **(c)** $TE_{4,5}$ mode and **(d)** $TE_{9,3}$ mode.

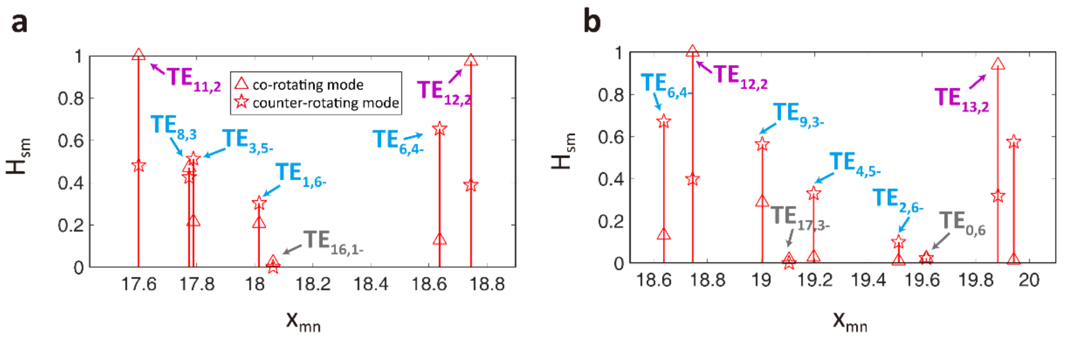

Figure s3. Comparison of coupling coefficients. The normalized coupling coefficients versus eigen values of different modes. Only rotating modes with higher coupling coefficients are pointed. The pink are operating modes, the blue are threatening competing modes, and the grey are weak competing modes. The counter-rotating mode is with a minus while the co-rotating mode has no specific sign.

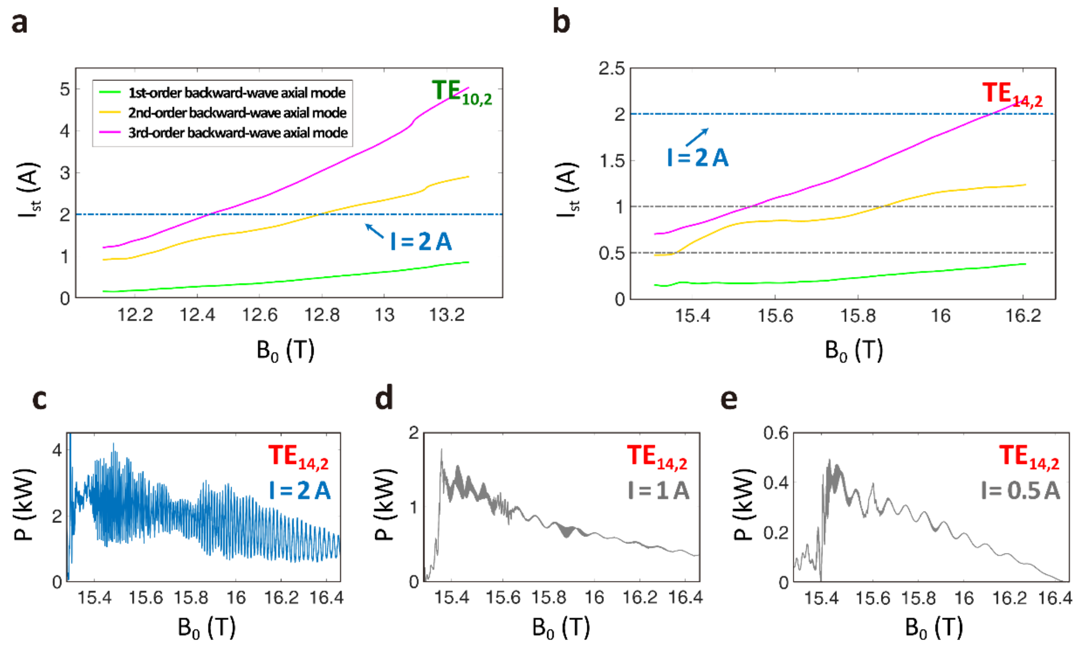

Figure s4. Start-oscillation current curves and $TE_{14,2}$ mode output powers. The start-oscillation current curves of **(a)** $TE_{10,2}$ mode and **(b)** $TE_{14,2}$ mode in different-order backward-wave axial-mode conditions. The output power of $TE_{14,2}$ mode during a linear magnetic field tuning process with a same sweeping rate when working currents become **(c)** I = 2 A, **(d)** I = 1 A and **(e)** I = 0.5 A, respectively.

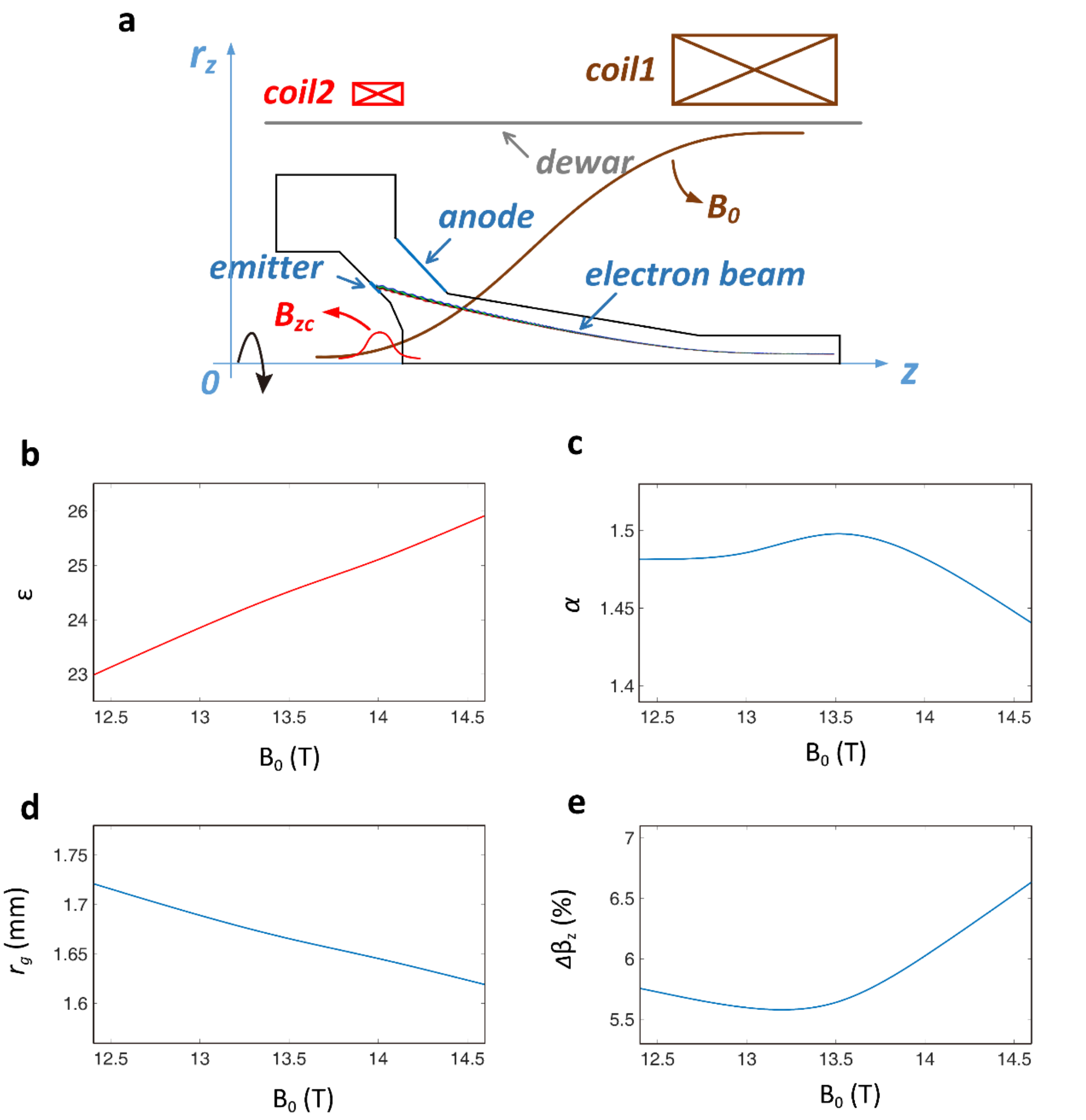

Figure s5. Variation of electron beam parameters in magnetic tuning. (a) A quarter section of the schematic diagram of electron gun attached with coil 1 and coil 2, as the primary coil and the auxiliary coil, respectively. Specific variation of **(b)** optimized magnetic compression ratio $\varepsilon = B_0/B_{zc}$ of system and corresponding electron beam parameters including **(c)** pitch factor α, **(d)** guiding center radius r_g and **(e)** velocity spread $\Delta\beta_z = \Delta v_z/v_z$, in primary magnetic field tuning.
